# “You cannot just stop life for just that”: a qualitative study on children’s experiences on refugee journey to Sweden

**DOI:** 10.1007/s00787-024-02387-w

**Published:** 2024-02-15

**Authors:** Erica Mattelin, Natalie Söderlind, Laura Korhonen

**Affiliations:** 1 Barnafrid, Swedish National Center On Violence Against Children, Department of Biomedical and Clinical Sciences, Linköping University, Linköping, Sweden; 2 Center for Social and Affective Neuroscience and Department of Biomedical and Clinical Sciences, Linköping University, Linköping, Sweden; 3 Save the Children, Stockholm, Sweden; 4 Department of Child and Adolescent Psychiatry and Department of Biomedical and Clinical Sciences, Linköping University, Linköping, Sweden

**Keywords:** Refugee, Adversity, Resilience, Agency, Human rights, Thematic analysis

## Abstract

Children with refugee backgrounds are often exposed to violence and other adverse experiences with well-known detrimental consequences on mental health. However, the current group of approximately 40 million child refugees is heterogeneous, stressing the importance of first-person perspectives in understanding children’s unique experiences and needs related to the migration process. Identifying contextual factors promoting health and resilience is also essential. For instance, the roles children play as active agents in constructing their own lives and adapting to different environments are poorly described in contemporary research on child refugees and their mental health. To address these knowledge gaps, we conducted qualitative interviews with a reflexive thematic analysis with eighteen children with refugee backgrounds in Sweden. This resulted in two main themes: *Longing for a good life that cannot be taken for granted* and *Challenged agency and changing rights.* The narratives indicate that children, although exposed to different challenges in different migration phases and based on gender and asylum status, have experiences of ordinary childhood with a desire for a good life with prospects. The results also show that children execute active and adaptable agency that is impacted by various factors. Reaching the full age appears to be a confusing and ambivalent transition due to changes in rights. The results pinpoint several possibilities to address factors that pose a risk to health and restrictions of rights among child refugees.

**Supplementary Information:**

The online version contains supplementary material available at 10.1007/s00787-024-02387-w.

## Introduction

Approximately 100 million people are forcibly displaced worldwide, half of whom are children [1]. The heterogeneity among this group is significant, for example, regarding the sociodemographic background and formal statuses. Reasons for leaving one’s country include war, persecution, human rights violations, famine, or natural catastrophes due to climate change [2].

The global situation has also impacted Sweden, which received 780,215 asylum claims from 2000 to 2022 [3]. Notably, in the last decade, the country became a destination for one of the highest numbers of asylum-seeking children, with 244,857 individuals aged 0–17 arriving between 2012 and 2022 [4]. Between 2000 and 2021, a significant portion (45%) of all (children and adults) asylum seekers in Sweden originated from Syria, Afghanistan, Iraq, and Somalia [5]. However, the high influx of asylum seekers during 2014–2015 led to changes in Sweden’s migration policy and to the implementation of restrictions on migration [6]. This was an apparent change from Sweden’s earlier inclusive migration policy. Although Sweden has had a historically inclusive migration policy, it is important to note that it has not been without challenges. There are several areas where difficulties have been described. Legally, children have the right to all kinds of health care, while those over 18 only have the right to care that cannot be deferred [7]. Children also have the right to education but are not legally obliged to attend. However, even though children have formal rights, there have been several challenges, such as access to mental health care for children with both refugee and migration backgrounds [8], difficulties with housing [9], and discrimination [10]. Beyond the documented difficulties, ongoing legislative changes have created an atmosphere of persistent uncertainty for individuals who arrived in 2014–2015. These individuals live with uncertainty regarding their permission to stay, as well as the conditions under which they are permitted to remain [11].

International migration encompasses the process of leaving the home country and settlement and integration into the society of the destination. The journey often includes short stays with repeated interruptions in human relationships. Exposure to adverse experiences, including violence, is usually significant even if the migration process does not always necessitate complicated and dangerous journeys [12]. The consequences of exposure to adversities among children with refugee backgrounds are well documented and include mental health problems [13–15]. Concomitantly, data indicate different groups among children with refugee backgrounds**,** including well-adjusted individuals and groups with good health and those with severe problems, multimorbidity, and functional impairments [16, 17]. Different pre- and post-migratory stressors, such as lengthy procedures of seeking asylum and threats of repatriation, also contribute to the higher risk of mental distress [18]. Qualitative studies have provided empirical data on subjective experiences of mental distress and seeking support during the resettlement [19, 20].

Children with refugee backgrounds are often labeled passive and vulnerable victims dependent on adults [21, 22], even if we know that many individuals can adapt and integrate into a new environment despite adversities [23, 24]. Resilience [25] and agency—“an individual’s intrinsic capacity for intentional behavior developed within the individual’s environment(s) and subject to environmental influences” [26]—play a role in adaptation to new living conditions and maintaining mental health [27]. The concept of agency is often neglected in discussions on children with refugee backgrounds, even if US studies on unaccompanied migrant children have shown that children express their agency through multiple forms of assertions [26].

Previous qualitative research in Sweden has examined various aspects of well-being and acculturative stress in families with refugee backgrounds. Notably, the agency of children has been identified as a factor influencing the well-being of parents [28]. Other studies have explored how accompanied female refugee minors describe their faith and religion [29], the role of language [30], and how undocumented children actively contribute to protecting the family’s status [31]. Additionally, there has been research on the experiences of living in and leaving residential care [30, 31] and the school [32].

However, most of these studies have been restricted to different language groups and unaccompanied children. Few studies have provided an examination of the experiences of children with refugee backgrounds in general.

Children with refugee backgrounds have a rich spectrum of experiences endorsing qualitative approaches to understanding their perspectives and needs. In this study, we addressed the following research questions:

- How do children with refugee backgrounds describe their experiences from different phases of the migration journey and construct their lives and agency?
- Does time-, gender-, or asylum status-related factors impact the experiences?

The study focuses on children who have recently arrived in Sweden, within the last 18 months. It aims to capture the shared and varying experiences related to the migration journey and the initial resettlement phase. The results are discussed regarding the rights of a child, agency, and the perspectives of exposure to adversity, mental health, and resilience.

## Methods

A detailed description of the methods is provided in the Supplementary information.

### Study design

This study is a sub-study in the ongoing prospective longitudinal *Long way to shelter*—research project [33]. This qualitative study used in-depth interviews and reflexive thematic analysis to investigate the experiences of children with refugee backgrounds [34–36]. This study is presented according to the COREQ-standard [37].

### Participants and recruitment

We recruited participants fulfilling the following inclusion criteria: 15–17 years at the time of the recruitment, refugee background, and had claimed asylum in Sweden a maximum of 18 months before the interview date [33]. The recruitment was done across Sweden through health care, social services, social media, and civil society, such as non-governmental organizations working with the target population. The researchers subsequently contacted those who had agreed to participate to obtain informed consent and schedule an interview. All information was given in oral and written forms in the participant’s native language. The study participants were eleven girls and seven boys from Bosnia, Syria, Iraq, Iran, United Arab Emirates, Somalia, Eritrea, Gambia, Uganda, Nigeria, Sudan, and Peru and had mixed asylum statuses.

### Data collection

An interview guide was designed with a few overarching questions concerning the experiences in the home country, issues related to the entire migration process, life during the first year in Sweden, and children’s future aspirations for the ten years ahead. We used semi-structured, authorized translator-assisted qualitative interviews and the “Teller-focused interview” method [38]. The interviews were conducted during April 2019 and February 2021 and were approximately 60 min long. They were audio-recorded and transcribed verbatim.

### Data analysis

Interviews were analyzed using NVivo Qualitative Research Software (version 12) and reflexive thematic analysis with inductive and latent constructionist approaches [34, 35]. The six-step method [34] and trustworthiness criteria [39] were used to assure the stringency in the analysis. Each interview was treated with equal attention in the analysis. We also analyzed the data based on time, asylum status, and gender. Direct quotes are used to illustrate the results. Sometimes, sections with long texts are shortened due to translation-related issues, such as repeated answers. This is marked with (…) in the text.

## Results

Two major themes were identified in the data (Table 1). The first theme, “Longing for a good life that cannot be taken for granted,” describes children’s experiences of trying to live their lives under several challenges. The second theme, “Challenged agency and changing rights,” comprises how children use their agency during different phases of agency and how various factors impact this. The theme also describes the impact of changing rights when reaching the full age.

**Table 1:** Themes and sub-themes

Themes	Sub-themes
Longing for the good life that cannot be taken for granted	Experiences of an ordinary childhood
	Challenging factors
Challenged agency and changing rights	The agency is being tested
	Reaching the full age can change everything

The results are presented as themes and sub-themes with illustrations of migration phase-specific differences regarding the experiences. In addition, the impact of age, gender, and asylum status on sub-themes are reported (Supplementary Table 2).

## Theme 1: Longing for a good life that cannot be taken for granted

**The first theme has two sub-themes** **:** ***Experiences of an ordinary childhood and Challenging factors.***

### *A) *Sub-theme:* experiences of an ordinary childhood*

The sub-theme describes experiences related to (a) hobbies and leisure time activities, (b) school and education, and (c) organizing everyday life.

Hobbies and leisure time activities:

Several interviewed children said that they had experienced, to a certain extent, an ordinary childhood and everyday life with significant social relationships, activities with friends, and various hobbies before fleeing. Children also tried to keep up with age-appropriate activities, make friends, and live as ordinary life as possible during migration. In many cases, siblings were important in maintaining an active life. One participant described her experiences before fleeing as follows:

*It was a normal childhood the whole time I was with my mom, and my father worked, and then he came in the evening home (...) I wrote my diary; I loved to sing and dance. Sometimes my siblings and I went shopping or drank coffee, which was our free time for watching tv shows.*

Attending school had been a priority for most of the children in all migration phases. Some explained that they could not attend school regularly in their home countries while fleeing. Once in Sweden, several children described a willingness to return to school, start learning Swedish, and meet peers. The school also facilitated independence and offered the possibility to learn about society and take up hobbies. Some children said that waiting for school to start due to the summer break was devastating. They indicated that everyday life and routines came back when school began. For example, one boy described how his life was much more balanced after returning to school.

*I only went to school when we could afford it, but when I came here to Sweden when I was fourteen, life got more balanced.*

The attitudes toward school were generally positive. Children dreamt of studying and acknowledged the opportunity for free schooling. Many envisaged a future in Sweden and wanted to stay, while others described Sweden as a transient stop on their journey to other countries or back home. Many children expressed a will to contribute by supporting the family and paying back for the sacrifices that have enabled the possibility of a better future, open options, and a life with fewer worries. One boy expressed his hope for a promising future:

*The idea that I should become a doctor is good for me, and I should support my family.*

Organizing everyday life:

Some children had family members or friends already living in Sweden. This helped preserve one’s culture and language, enabling a feeling of continuity. Many appreciated that their basic needs, such as food and health care access, and resources were better supplied. “*There is less stuff you need to worry about,*” explained one child.

Time-, gender-, and asylum status-based analysis of the sub-theme:

As Supplementary Table 2 shows, the description of experiences related to hobbies and leisure time activities did not show any significant changes between different phases of migration, including analysis of the impact of being six months vs 18 months in Sweden, nor based on gender or asylum status. Regarding experiences related to school, a shift from a wish to participate in school (in the home country and while fleeing) to a wish for a future as an educated person (while settled in Sweden) was identified without any apparent gender- or asylum status-related differences. The description of organizing everyday life focuses on the situation in Sweden and has no gender or asylum status-specific elements.

### B) Sub-theme: challenging factors

The sub-theme describes experiences related to (a) exposure to different kinds of adversities, including violence, (b) family separations, (c) language difficulties, (d) mental health challenges, and (e) difficulties in integrating.

Exposures to adversities:

The narratives contained many difficulties, and life appeared to be a continuous navigation between a desire for everyday life and obstacles. Experiences of poverty in home countries and poor housing in Sweden were evident in many stories. A qualitative change from absolute poverty in terms of lack of food and access to school in home countries to relative poverty in Sweden was evident (Supplementary Table 2).

Children described not getting their rights, such as freedom, justice, and gender equality, fulfilled. For girls, gender-based violence and oppression appeared as a challenge. For example, one of the girls explained that she and her family chose to leave because of the lack of freedom and gender oppression in her country of origin. She described that this affected everyday life in terms of what you could wear and the opportunities as a woman. Violation of rights and exclusion from society were reasons why another participant was forced to flee, even if the living conditions in the country of origin were otherwise good.

*We used to live in a villa and had everything we wanted (…) But I know that we came here to live a simpler life, and a better life, to have a better future.* And she continued: *It’s like considered not a local (…) if a foreigner, like me, for example, if I don’t get good grades if I’m acting- if I have bad behavior, they just punish me.*

Children also experience threats and violence in their families, schools, and society. Significantly, the experiences from the migration journeys indicated various risks and frequent incidents of direct and witness violence. The stress of having correct documents, the arbitrariness of the administrative systems, and the unknown waiting time were shared by all interviewees. Several participants had traveled with peers or previously unknown adults in the hands of smugglers. One participant described his experiences of being kidnapped:

*We were imprisoned for a year in the underground basement, and we were there for two years, and we were only fed once a day, once a day. The only food we got was macaroni.*

Family separations:

The family is central in almost all narratives (Supplementary Table 2). The experiences vary but did not show any apparent gender-based differences except for boys being more frequent in the group of unaccompanied minors. Some children had experienced family separation already before fleeing, while others had nuclear families. During the migration and while in Sweden, the families could be separated or reunited; new family constellations were established. Both good and bad experiences were evident.

Children told how one of the parents, often the father, had fled before the rest of the family. This was a difficult situation to cope as explained by one child:

*Then I lived with the hope of seeing him again, that hope that gave me the strength to endure that period without him then. I lived in the hope of seeing him again; I evoked many memories of him.*

In some cases, both parents had fled, and the children had been left to grandparents or other relatives. Separations could be several years long with limited contact. Some parents had established new lives and families in resettlement countries. This was a significant uncertainty factor for the children, expecting to be united with their parents. A boy described his complicated experiences:

*And then I got to stay with grandma, which wasn’t good, and she said I can’t talk to my mom (…) we had two twins; one was sick and passed away later.* (*...) the other twin who survived, mom left to aunt. And my dad wasn’t there either, so he went to* [country] *because Dad didn’t want us to go to Mom. And he picked us up to* [country] *(…). So when he had us, he wanted to get married, and I didn’t want him to get married.*

Some children had lost both of their parents and even close relatives who had previously taken care of them. As explained by one boy, no other alternatives were left except to accept the situation.

*I only have my brother as a family. I had grandpa at first, as I just told you, but he has died.* He continued to explain: *Yes, my father, I never got to know. My mother, she disappeared from one day to the next, just. It’s sad, of course, but what to do? Life can’t just -you cannot just stop life for just that.*

The situation for the unaccompanied seemed very challenging during the migration journey and while in Sweden. In this group, narratives suggested an essential role for friends as a substitute for family members.

Language difficulties:

Upon arrival, lack of competence in Swedish was challenging everyday life. Some children did not recognize themselves when language became a sudden barrier to society and social contacts. While acknowledging the importance of learning the Swedish language, the children used social media to keep in touch with old friends or to meet people from the same language group. This gave them the possibility to communicate unhindered. One girl described her experiences:

*I couldn’t speak Swedish at all. I could speak English, but I didn’t use it either. I was ashamed to speak English (...) I just went home from school and had nothing else. At first, I was a little depressed because I felt the difference, which means that in* [country]*, I used to communicate with everyone, and I had many people around me.*

Mental health issues:

Some children described feelings of social isolation, being abandoned by old friends, and lacking behind others because of the time taken by the long migration process. The children also expressed sadness, grief, and homesickness; they missed friends and relatives. Life had not necessarily become better. Some narratives were ambivalent and full of confusion regarding the reasons to flee and a new life in Sweden, which sometimes felt like a pale copy of the previous life. While longing for stability and permanent solutions, the understanding that the stay in Sweden was not only a brief visit came gradually. Children’s and adults’ perspectives were not always harmonious, as exemplified in the following excerpt:

*Parents are like you’re at the best age for moving here. And they’re right, but it doesn’t make it that much easier. It’s still so hard. And my cousin, she’s seven years old, and everybody in my family is like “oh my god, she’s so lucky, she’s seven, so she’s going to live in Sweden, she’s going to grow up there, she’s going to be so lucky”. (...) so she started going to school, and she didn’t know English, so she couldn’t understand anything people were saying. She had problems finding friends and didn’t like to go to school.*

Sometimes, problems with sad feelings, sleeping difficulties, and other mental issues had begun in the country of origin. Arriving in Sweden could result in a worsening of well-being or the opposite. Children were, however, able to express both gratefulness and grief simultaneously.

Difficulties in integrating:

Yet another sub-theme in children’s narratives on challenging factors was difficulties and experiences of being excluded. Life in the new country was not entirely under the same conditions. They also described the ambivalent reception of refugees: a refugee is considered a foreigner, vulnerable, and at the same time, the experience was that Swedish society sets high demands on the newly arrived. The children explained the paradox of being dependent on the system but lacking information on how to contact authorities to get help. According to some children, living in a neighborhood with high immigrant density was experienced as more accessible and safer.

Time-, gender-, and asylum status-based analysis of the sub-theme:

The challenging factors showed differences in different phases of the migration journey, but no clear differences were found in the analysis of the  impact of being 6 months vs 18 months in Sweden. Girls described gender-based violence and oppression in their home countries, and the unaccompanied minors described the most severe forms of violence. In Sweden, the children stop describing violence in terms of physical violence. Instead, they described racism, discrimination, polite exclusion, and bullying. For example, some children experienced how people were favored or grouped by country of origin or based on skin color.

Based on the narratives, obtaining skills in Swedish was a central topic and appeared as a key for integration and, at the same time, as a post-migratory stressor. Experiences regarding family separations varied greatly in different migration phases and individuals. No clear differences based on gender or asylum status could be identified regarding language difficulties, mental health issues, or integration (Supplementary Table 2).

## Theme 2: Challenged agency and changing rights

**The second theme has two sub-themes: (a) The agency is being tested, and (b) Reaching the full age can change everything.**

### A) Sub-theme: The agency is being tested

According to the narratives, it is evident that children with refugee backgrounds use their agency—different intentional behaviors—to influence their own and others’ lives. At the same time, several factors in the environment where children in different migration phases find themselves impact the agency and can facilitate or restrict it (Supplementary Table 3). This was exemplified in decision-making regarding fleeing. Sometimes, the children did not tell their parents about their intentions to leave. For example, one boy explained the decision he made at the age of ten years:

*It was my decision; I wanted to escape across the border. All my childhood friends and acquaintances had fled the country, so I wanted to do it myself.*

Further, he explained crossing over his country of origin by foot, not knowing anyone, but that he learned to know people on the migration journey. A trip that took over six years. He described how he had survived with the help of other refugees with a high motivation and intention to come to Europe:

*I didn’t have anyone I know of, but you met during the trip.* He continued: *I had a tough time and had difficult times, so everyone felt sorry for me. The prisoners managed to collect 500, and a friend of mine who lived in* [country]* managed 500, a total of 1,500. I paid them* [kidnappers] *(...) and I managed to get to the Red Cross.*

Many children explained being part of the decision-making but having less autonomy. One of the girls explained that her father had asked her beforehand. Still, she also mentioned that they did not have a choice because of her father’s difficult situation in their country of origin:

*I’ve been a part of the decision. He* [father] *asked us one by one, “what do you think, do you want to come with to Sweden?”. And then we gave our opinions on that, but I must emphasize that it was not a choice. He* [father]* could not stay there anymore, and he had to leave.*

The involvement in the decision-making might be seen as a paradox since it is evident that the children are aware that the only or best option is to leave their country of origin due to existing circumstances. It is also important to acknowledge that not all the children could express their involvement in the decision to leave their country of origin. *I didn’t know what Sweden was or any other country. It just turned out that way; they left us here*, explained one participant. Even when not involved, children showed the capability to see why the decision was made, as told by a boy:

*It was not me who decided to leave, but there is no justice in my country; everything is problematic—so we decided to go.*

In some cases, children’s agency was restricted to protect them. One participant described:

[Militant group]* was in the area and controlled it, so we hid. And they didn’t know that we were going to Sweden. It was a secret.* She continued: *You didn’t discuss this openly with anyone. Mom and grandma knew about all this information, and it was just the two of them.*

The restricted agency was also described by a 17-year-old girl. She had been placed by social services at a foster home. In this case, the social services deprived her of the possibility to move and did not listen to her wishes.

*I say I want to go out. I want to meet my friends. I want to sleep over with them. “No, you have to talk to your assigned legal guardian”, who says no, and the social services say you are a child. So I become sad, and I cry, and I do nothing. I keep it to myself.*

She also described the problems related to out-of-home care. While clearly describing her compromised agency and the lack of a child perspective, she also explained how she handled this problem.

*The problem is that I do not understand why I was placed with this family who cannot speak Swedish, does not know the system, and does not care about me. I would rather live alone and take responsibility for my studies.*

Time-, gender-, and asylum status-based analysis of the sub-theme:

Before fleeing, children mainly describe agency in relation to the decision-making regarding the flight process. During the migration, there seem to be few other options than continuing the journey. In Sweden, the agency is described in terms of adaptation, such as by taking advantage of the opportunities given. The children identify several factors that test the agency in different migration phases, including restrictions made by authorities (Supplementary Table 3).

### B) Sub-theme: Reaching the full age can change everything

In the children’s narratives of their journey, we also identified a significant change regarding the transition from childhood to adulthood. Reaching the full age at 18 is filled with ambiguities. The concerns were mainly related to the shift in rights and regulations depending on the age. For example, the Convention on the Rights of a Child (CRC) [40] is incorporated into the Swedish national legislation, implying that other laws must be interpreted based on the CRC. This provides a robust framework where a child’s best interest guides decision-making, including the right to stay in Sweden.

Some children described the 18th birthday as the primary stressor and worry. Concerns about the asylum process, such as reunification with parents and siblings, were especially evident. One participant explained that he could not reunite with his mother because he had reached full age while living in Sweden, even though he applied for reunification when he was 17. He described this as being deprived of the right of reunification. He told how this had affected his health and ability to function.

*When I applied* [family reunification], *I was under 17 (…). But then (…) they said that I’m 18 and must meet specific requirements, criteria with work and housing and stuff. This has affected me. Well, I cannot go to school and concentrate.*

Another girl described a similar experience where her 20-year-old sister was left behind in one of the transient countries because “*she has no hope of traveling”* and another because she had turned 18 years the day the family got their decision placement in Sweden.

*When we received the papers on* [date]*, we discovered that* [sister] *was omitted. We knew they were taking her out of our family because she had become an adult. This shocked us.*

A 17-year-old boy described how clear the division between child and adult could be. He had arrived with his brother, who was still a minor. This meant that they lived with other minors and had the right and opportunity to study. After turning 18, his brother was forced to move away and start working in the hope of receiving a work permit.

*But my brother did not have much time left before turning 18. And I remember that we lived in a house in* [country]*, where many boys lived (…), and my brother and I lived there, from June until September. Then he* [brother] *had to move because he turned 18.*

While reaching the full age appeared to be a curse for some children, others were looking forward to obtaining legal rights to control decisions and govern themselves. In these cases, children described problems related to being a minor that restricts them in the Swedish system. One girl described this by saying that she had been moved from one foster family to another without being consulted, even if she had both wishes and own ideas on how to solve the situation:

*I did not want to move from this family; I said I felt comfortable with them, but they did not listen to me, refused to let me live with them, and moved me.*

Time-, gender-, and asylum status-based analysis of the sub-theme:

We did not observe significant differences based on gender. However, the theme is practically non-existent before deciding to leave one’s home country. Once the decision to leave is made, the 18th birthday becomes one of the determining factors for whether one accompanies the family or not. In Sweden, after migration, this theme becomes particularly evident. Here, the children describe how it affects both their freedom of movement and living situation, based on restrictions when under 18, as well as issues related to older siblings having to move from open residential homes and reunification not being possible for others.

## Discussion

This study provides empirical knowledge of the migration journey-related experiences among a rich sample of children with different refugee backgrounds in Sweden. The data have been analyzed regarding time, gender, and asylum status.

The first main result is that although exposed to various challenges, the main narrative was not being a victim. The children acknowledged that they had everyday life experiences and memories of a rather ordinary childhood and a wish to live a good life with prospects. This narrative was shared by all children regardless of background, gender, or asylum status. The narrative was an essential foundation for life in the resettlement country and was a reference point for further aspirations. One of the highest dreams was a desire for a good life with opportunities to study and contribute. Having self-perceived experiences of a normal childhood and a longing for everyday life may help maintain a sense of continuity and predictability even during changing environments and social contacts sustained over long periods. The generalizable and shareable experiences may also facilitate a more stable sense of belonging in the new social milieu, which is important for well-being, satisfaction, and other positive outcomes [41, 42].

There are no previous Swedish qualitative studies reporting similar results, but a wish for a normal life was previously reported in an Icelandic study on young adult refugee men [43]. In this study, the refugees described the importance of having a structure and routine in their lives and emphasized a sense of doing something worthwhile. These results align with previously reported adverse effects of waiting time for an asylum decision and lack of opportunities, including, e.g., low self-esteem, lack of control, and a feeling of not belonging with associated mental health problems [43–45].

Based on the obtained results in this study, schools play an important role in everyday life and routines. Some children pointed out that waiting for school to start after a months-long summer break was devastating, while going to school was empowering and enhanced optimism regarding prospects for future opportunities. This is understandable because many child refugees have little or interrupted education because of the flight and societal problems in their country of origin [46]. Several previous studies have recognized the role of schools and host country language proficiency in supporting integration, social participation, building societal networks, and promoting resilience [47]. Significantly, the role of schools in promoting adjustment can be strengthened by legislation and other regulatory frameworks, including policies to support the resettlement [48].

The second key finding is the pleiotropy of factors that challenge the possibility of an ordinary childhood among children with refugee backgrounds. Experiences of violence were common and had taken place in different migration phases. As shown in previous studies, exposures among unaccompanied children were the most frequent [49]. Interestingly, the descriptions of types of violence changed from physical violence in home countries to bullying, exclusion, racism, and discrimination in Sweden. Apart from exposure to violence, one of the most potent narratives—shared through the interviews—was the separation from caregivers and the split of families, as also reported previously by [50]. Family separation due to the death of caregivers is not uncommon either [51] and was also present in the narratives. As shown in a recent systematic review [52], child refugees described a deep sense of loss. Separation from the caregivers is also a known barrier to settlement and risk of marginalization and inequalities [52]. This and the insufficient societal capacity to secure the best interest of the child were also illustrated in some narratives. Legal and procedural barriers also hinder family reunification, causing guilt, worry, distress, and other mental problems among child refugees [50, 53]. Many of these factors did not show any gender- or asylum status-related differences, but due to the limited number of interviews conducted, more studies are warranted.

Despite the identified challenges, children with refugee backgrounds appear resourceful with a clear vision of a positive future. The results support previous studies on resilience strategies [54]. These comprise acting autonomously, performing at school, perceiving support from peers and parents, and participating in the new society. Additional resilience and protective factors include self-esteem, maintenance of cultural identity, safety, and innovative social care services stressing the importance of policies and societal approaches such as inclusive society [23].

The third main finding shows how children with refugee backgrounds use their agency in different situations. Similar to previous studies on agency among unaccompanied minors [26, 55], children with refugee backgrounds who participated in this study had personal rationales that motivated their decisions. The narratives also indicated how the agency could be restricted or facilitated. As shown earlier, enabling the agency often occurred within the family or social networks that aided and supported children in different migration phases [26]. Assertion of the agency, such as a willingness to pay smugglers to reach Europe and pragmatic dependence on co-migrants, was also evident in some narratives.

The fourth main finding is that reaching the full age is a particularly ambivalent transition with changed legal rights and responsibilities. This aspect is generally less documented in the research literature. Still, there are indications that young adults forcibly returned to Afghanistan face violence, destitution, and barriers to entering education or employment, as well as mental health problems [56]. In Sweden, turning 18 affects the legal status and asylum and family reunification processes. For some children with refugee backgrounds, the full age was a distressing and uncertain period, as previously indicated [57]. Some participants, however, eagerly waited for the termination of the control executed by the assigned legal guardians. The transition from a refugee minor to a young adult refugee seems a critical topic warranting more studies.

The current definition constructs being a refugee as a human right and emphasizes nations’ responsibility to protect the stateless [58]. When analyzing the obtained results from this perspective, it is evident that the narratives describe how children’s rights are violated at all phases of the migration process. For example, family reunification problems violate a child’s rights as the CRC Article 10, and several children described a lack of protection breaking Article 19 [40]. Protective measures that help child refugees maintain good mental health and the ability to participate should be paid more attention to in line with CRC Articles 24 and 27.

### Strengths and limitations

The small sample size of this qualitative study limits the generalizability of the findings across different refugee populations and age groups. The findings regarding time-, gender-, and asylum status-related factors especially warrant more studies. Various political and cultural contexts uniquely impact children’s experiences, and thus, the results related to resettlement should be interpreted in the societal context of Sweden.

This study used teller-focused interviews and the perspective of a child’s agency, making the interview process more reciprocal. However, the discussions were translator assisted, which might have introduced a bias, for example, due to misinterpretations, lack of nuances in translations, and technical issues in telephone translations. Communication using the children’s native language would have been ideal, but this was not feasible due to logistical limitations.

## Conclusions

We conclude that a shift from a negatively biased view of child refugees to a more nuanced view that simultaneously embraces children’s rights and vulnerabilities and their resilience, competencies, goals, and strengths is warranted. Keeping with the risk for adversity with known negative consequences and understanding that adolescent development continues well into the mid-20 s [59], developmentally appropriate framing of laws and policies should be initiated. For example, the age limit for temporary residency permits granted as minors could be adjusted from 18 to 24 years. Also, full access to health care services and appropriate social support should be guaranteed during young adulthood. Participatory approaches might help develop services based on individual needs and with respect for the rights of a child.

## Supplementary Information

Below is the link to the electronic supplementary material.

Supplementary file1 (DOCX 46 KB)

Supplementary file2 (DOCX 32 KB)

## Data Availability

The ethics approval was obtained for public sharing and data presentation on the group level only. The data can only be used for the approved research and cannot be shared by the authors.
