# Supplementary material for: “You cannot just stop life for just that”: a qualitative study on children’s experiences on refugee journey to Sweden
Source: Eur Child Adolesc Psychiatry. 2024 Feb 15;33(9):3133–43. doi: 10.1007/s00787-024-02387-w (PMC11424661; doi:10.1007/s00787-024-02387-w)
Supplement: Supplementary file 1 — Supplementary file1 (DOCX 46 KB) [file 787_2024_2387_MOESM1_ESM.docx]

**Supplementary information**

**Detailed description of the methods**

*Definitions*

Children with a refugee background:

There is no universally agreed-upon terminology for displaced children in the scientific literature. In this study, the term *children with a refugee background* (“barn och unga på flykt” in Swedish) refers to asylum-seeking children who have not received decisions on their claim, children with a formal refugee status according to the Geneva Convention [1, 2], children with status as in otherwise need of protection in the Swedish legal system, quota refugees (ie, a person who has been selected by the UNHCR to be resettled to a third country), children who have been reunited with their families who have granted asylum in Sweden and children who have been declined to stay but have not been able to leave the country [3].

Mental Health:

In this study, we apply the WHO definition of mental health [4], stating that “Mental health is a state of mental well-being that enables people to cope with the stresses of life, realize their abilities, learn well and work well, and contribute to their community.” The definition underlines that mental health is more than the absence of mental disorders and that it exists as a continuum, can vary over time, and is experienced differently from one person to the next. The WHO definition also pays attention to participation in society.

Agency:

Agency refers to “an individual’s intrinsic capacity for intentional behavior developed within the individual’s environment(s) and subject to environmental influences” [5]. In view of this, a child can be considered an agent, but the agency is influenced – restricted and facilitated - depending on the social, political, and legal contexts.

*Study design*

This study is a sub-study in the ongoing prospective longitudinal *Long Way to Shelter* – research project [3]. This qualitative study used in-depth interviews and reflexive thematic analysis to investigate the experiences of children with a refugee background [6-8]. The study is presented according to the COREQ-standard [9].

*Participants and recruitment*

We recruited participants fulfilling the following inclusion criteria: 15-17 years, refugee background, and had claimed asylum in Sweden a maximum of 18 months before the interview date [3]. It should be noted that one child turned 18 years of age after recruitment.

The recruitment was done across Sweden through health care, social services, social media, and civil society, such as non-governmental organizations working with the target population. The researchers subsequently contacted those who had agreed to participate in order to obtain informed consent and schedule an interview. All information was given in oral and written forms in the participant's native language. The selection of the participants was conducted from consecutive sampling.

The study participants were eleven girls and seven boys born in Bosnia, Syria, Iraq, Iran, United Arab Emirates, Somalia, Eritrea, Gambia, Uganda, Nigeria, Sudan, and Peru and had mixed asylum statuses.

*Data collection*

An interview guide (see Supplementary Appendix 1) was designed with a few overarching questions concerning the experiences in the home country (e.g., school, friends, hobbies, well-being), issues related to the entire migration process (e.g., reasons to flee, memories from the journey), life during the first year in Sweden and children´s future aspirations for the ten years ahead.

The participants chose where the interview was conducted. During the COVID-19 pandemic, interviews were mainly conducted by online video calls. We used semi-structured, authorized translator-assisted qualitative interviews and the "Teller-focused interview" method, which is particularly suitable for studies on sensitive topics and ongoing processes [10]. The interviews were conducted by EM, NS, and two research assistants (Hania Kutabi (HK) and Sofia Michael (SM)) during April 2019 and February 2021. Depending on the choice of the interview location and availability, the translators were present in the interviews, or telephone translations were used. When possible, the translators were authorized translators according to the Swedish system for translators. However, this was not always possible due to the lack of authorized interpreters in some of the languages spoken.

The reason for having multiple interviewers was the caution in traveling during the pandemic. This left us with using the geographically closest interviewer to the participant. The interviews were approximately 60 minutes long.

All interviewers were female with a background as clinical psychologists (EM, HK, SM) and MSc in Child and Youth Science (NS), influenced by developmental psychology and childhood studies and trained in talking to and interviewing children. There was no prior relationship between the participants and the interviewers.

Before the interview, the participants were informed that the qualitative study was part of a broader research project. It was clarified that the intention was to gather the participants' subjective experiences, and the participants were encouraged to talk openly. The interpreters were instructed on the importance of strictly interpreting only what is said. They were also encouraged to disclose insecurity regarding translation to discuss proper re-wording.

Interviews were audio-recorded and transcribed verbatim. A random sample of 20% of the interviews (N=4) was quality assessed by listening to the discussions and cross-checking them with the transcripts. We did not return transcripts to the children. The interviews were anonymized by removing names and identifying features. Field notes were taken, and the interviews were discussed in the research group. The recorded audio files and transcripts were archived in the Linköping university’s digital secure file system with a password to which only the authors had access.

*Data analysis*

Interviews were analyzed using NVivo Qualitative Research Software (version 12). The data was analyzed using reflexive thematic analysis with inductive and latent constructionist approaches [6, 7]. The six-step method [6] and trustworthiness criteria [11] were used to assure the stringency in the analysis. Each interview was treated with equal attention in the analysis.

All authors became familiar with the data. No pre-defined codes were used. EM, NS and LK coded the first interview. The rest of the interviews were coded and discussed by EM and NS. Categories and themes were created iteratively by EM, NS, and LK. In each theme, a central organizing concept was identified. Themes were discussed with all authors, and the different phases of the analysis process were repeated to check the themes against the original data. The final themes were non-overlapping and had a clear relationship with each other. Supplementary Table 1 shows an example of the data analysis. Direct quotes are used to illustrate the results. In some cases, sections with long texts due to translation-related issues, such as repeated answers, are shortened. This is marked with (…) in the text.

*Subanalysis*

Lastly, we also analyzed the data based on time, asylum status and gender.

*References*

[1] UN, "Convention Relating to the Status of Refugees," *Treaty Series,* vol. 189, pp. 137-220, 1951.

[2] UN, "Protocol Relating to the Status of Refugees," *Treaty Series,* vol. 606, pp. 267-276, 1967.

[3] E. Mattelin, A. R. Khanolkar, F. Fröberg, L. Jonsson, and L. Korhonen, "'Long journey to shelter': a study protocol: a prospective longitudinal analysis of mental health and its determinants, exposure to violence and subjective experiences of the migration process among adolescent and young adult migrants in Sweden," (in eng), *BMJ Open,* vol. 11, no. 9, p. e043822, Sep 30 2021, doi: 10.1136/bmjopen-2020-043822.

[4] WHO, "Promoting Mental Health: Concepts, Emerging Evidence, Practice," 2005. [Online]. Available: <https://www.who.int/publications/i/item/9241562943>

[5] A. Thompson, R. M. Torres, K. Swanson, S. A. Blue, and Ó. M. H. Hernández, "Re-conceptualising agency in migrant children from Central America and Mexico," *Journal of Ethnic and Migration Studies,* vol. 45, no. 2, pp. 235-252, 2019/01/25 2019, doi: 10.1080/1369183X.2017.1404258.

[6] V. Braun and V. Clarke, "Using thematic analysis in psychology," *Qualitative Research in Psychology,* vol. 3, no. 2, pp. 77-101, 2006/01/01 2006, doi: 10.1191/1478088706qp063oa.

[7] V. Braun and V. Clarke, "Reflecting on reflexive thematic analysis," *Qualitative Research in Sport, Exercise and Health,* vol. 11, no. 4, pp. 589-597, 2019/08/08 2019, doi: 10.1080/2159676X.2019.1628806.

[8] J. Ringblom, M. Proczkowska, L. Korhonen, and I. Wåhlin, "Experiences of paediatric emergence delirium - from parents' and a child's perspective," (in eng), *Scand J Caring Sci,* Jun 22 2021, doi: 10.1111/scs.13011.

[9] A. Tong, P. Sainsbury, and J. Craig, "Consolidated criteria for reporting qualitative research (COREQ): a 32-item checklist for interviews and focus groups," *International Journal for Quality in Health Care,* vol. 19, no. 6, pp. 349-357, 2007, doi: 10.1093/intqhc/mzm042.

[10] M. Hydén, "The teller-focused interview: Interviewing as a relational practice," *Qualitative Social Work,* vol. 13, no. 6, pp. 795-812, 2014, doi: 10.1177/1473325013506247.

[11] L. S. Nowell, J. M. Norris, D. E. White, and N. J. Moules, "Thematic Analysis:Striving to Meet the Trustworthiness Criteria," *International Journal of Qualitative Methods,* vol. 16, no. 1, p. 1609406917733847, 2017, doi: 10.1177/1609406917733847.

**Interview guide**

*The guide below includes suggestions for questions that cover critical areas within the interview. It should be seen as a flexible guide encouraging adolescents to describe their experiences and expectations.*

**Introduction/orientation**

I will start the interview, but first, I will tell you how it will go. I will ask questions about you, both about you as a person and what you have experienced, how you feel, and how you have felt before. The purpose is that I want to hear your story and your experiences and feelings. There are no right or wrong answers. Sometimes I may repeat a question or ask you to develop and tell me more about something. If there is a question that you feel you don’t want to answer, that's fine. It is also ok to end the interview at any time. We will talk for about an hour, and I’ll record the conversation. The recording ensures we don’t miss or forget what we say today. Only me and my colleagues will listen to the recording. Do you have any questions for me before we start?

1. About you and the time before you decided to leave your country of origin.

### Tell me about you, who you are, your age, etc.

### What country are you from?

- How would you describe your ethnicity?
- How has your upbringing been until now?
- How would you describe your relationship with friends? How was your relationship with your family in your country of origin?
- Have you had access to schooling before you came to Sweden?
- How would you describe your schooling before you came to Sweden and after?
- What did you enjoy doing in your spare time in your home country?
- How would you describe your mental and physical well-being in your country of origin?

1. During the migration to Sweden
   - When was it decided that you should leave your country of origin?
   - What was the reason for you/your family leaving?
   - Did you know from the beginning that you were going to Sweden?
   - How did the journey go about?
   - When did you arrive in Sweden?
2. During the first time in Sweden (up to six months)

- How were your first days in Sweden? During the first days?
- How did you feel?
- What organization/authorities did you get in contact with?
- What persons did you meet with?
- How did you experience the reception when you arrived in Sweden?
- When did you start school?
- What do you think worked well?
- What worked less well?
- Did you miss something when you came to Sweden?

1. About being in Sweden right now (approx. 12 months)

- Tell me about how you live today (where you live, with whom, etc.)
- How do you enjoy being in Sweden?
- How is your contact with your family?
- How is your contact with your friends?
- Are you attending school?
- What do you enjoy doing in your spare time?
- How would you describe your mental- and physical well-being today compared with before you arrived in Sweden?
- How would you describe your mental - and physical well-being today compared to the first time in Sweden?
- Do you get any support or help right now from health care and non-governmental organization?
- Would you like to have any other support or help? How have you experienced the process of seeking asylum (care, law)?
- Is Sweden as expected? If you think back on the expectations you had?
- Think about others in the same situation as you were a year ago. What would you say to them?

1. About being in Sweden right now (approx. 12 months)
   - What will your life look like in 10 years (e.g., living in Sweden, family, etc.)?
   - What do you think it will look like in 10 years?
2. Is there something that I’ve forgotten to ask that you would like to add?

**Supplementary Table 1.** Examples of the data analysis process.

| Emerging code in the original text | Code | Sub-theme | Theme |
| --- | --- | --- | --- |
| Sometimes my siblings and I go shopping or drink coffee, this used to be our free time or watching TV shows  I used to play football with my friends | Age-appropriate activities | Experiences of an ordinary childhood | Longing for the good life that cannot be taken for granted |
| I used to play with my friends. So in (name of a country) I didn't sit at home all the time, but I was out a lot. | Peer relationships |  |  |
|  |  |  |  |
| When I came to Sweden, it was summer...the schools were closed…because I have two younger siblings who go to a preschool. Then when they started preschool in the fall, so mom also applied for us regular school. | Attending school |  |  |
| I want to study architecture, but I don't know if I can finish Swedish or not. But these studies take five years, and after five years I will work for three or four years, after which it is possible to study for the master. | Dreams |  |  |
|  |  |  |  |
|  |  |  |  |
