# Supplementary material for: “You cannot just stop life for just that”: a qualitative study on children’s experiences on refugee journey to Sweden
Source: Eur Child Adolesc Psychiatry. 2024 Feb 15;33(9):3133–43. doi: 10.1007/s00787-024-02387-w (PMC11424661; doi:10.1007/s00787-024-02387-w)
Supplement: Supplementary file 2 — Supplementary file2 (DOCX 32 KB) [file 787_2024_2387_MOESM2_ESM.docx]

**Supplementary Table 2.** Time, gender, and asylum-status-based analysis of the Theme of *Longing for a good life that cannot be taken for granted.*

| **Subthemes** |  | | **Pre-migration phase** | **Migration phase** | **Post-migration phase** |
| --- | --- | --- | --- | --- | --- |
| Experiences of an ordinary childhood | Hobbies and leisure time |  | e.g. play, soccer, friends, TV series | e.g. play, soccer, friends, TV series | e.g. play, soccer, friends, TV series, gym |
|  |  | Gender | - | - | - |
|  |  | Asylum status | - | - | - |
|  | School and education |  | Longing and wanting to participate in school | Longing and wanting to participate in school | Dreaming of higher education/being educated |
|  |  | Gender | - | - | - |
|  |  | Asylum status | - | - | - |
|  | Organizing everyday life |  |  |  | Access to food and health care better supplied. |
|  |  | Gender | - | - | - |
|  |  | Asylum status | - | - | - |
| Challenging factors | Exposures to adversities |  | Description of physical violence and oppression. Violence occurs in multiple arenas, such as in society, family, and schools.  Poverty, e.g. lack of food, access to school | During the migration, the violence is still physical but is also characterized by kidnapping and racism. New areas occur, such as by smugglers and police.  Relative poverty, e.g. poor housing | In Sweden, the children stop describing violence in terms of physical violence. Instead, they describe racism, discrimination, polite exclusion, and bullying.  Relative poverty, e.g. poor housing. |
|  |  | Gender | For girls, gender-based violence is a challenge. | - | - |
|  |  | Asylum status | - | The unaccompanied describe the most severe forms of violence. | - |
|  | Family separations |  | Very varying family constellations, nuclear and separated families,  forced to live with relatives. | Some families are separated (fully or partially), and some become reunited. Friends substitute for family. | Longing for reunion, sorrow for lost family, new constellations.  Reunion (successful or not) |
|  |  | Gender | - | - | - |
|  |  | Asylum status | - | The unaccompanied describe the most difficult situation. | The unaccompanied describe the most difficult situation. |
|  | Language difficulties |  | Not mentioned | Not mentioned | It is central to understanding the system, coping with school, belonging, and integrating with Swedes and other groups. Difficulties with the language make the children seek out their own language group. |
|  |  | Gender | - | - | - |
|  |  | Asylum status | - | - | - |
|  | Mental health issues |  | Sad feelings, sleeping difficulties, anxiety, fears, insecurity | Feelings of being abandoned by old friends; social isolation | Sadness, sleeping difficulties, grief, homesickness. Missing friends and relatives. Life in Sweden is not necessarily better. |
|  |  | Gender | - | - | - |
|  |  | Asylum status | - | - | - |
|  | Difficulties in integrating |  | - | - | Exclusion, ambivalent reception of the newly arrived. Dependent on the system but lacking information on how to get help. |
|  |  | Gender | - | - | - |
|  |  | Asylum status | - | - | - |

**Supplementary Table 3.** Time, gender, and asylum-status-based analysis of the Theme *Challenged agency and changing rights.*

| **Subthemes** |  | | **Pre-migration phase** | **Migration phase** | **Post-migration phase** |
| --- | --- | --- | --- | --- | --- |
| The agency is being tested |  |  | The children mainly describe agency in terms of having decided to flee or not to flee. | There are no other options than to continue the migration journey. | The agency is more often described in terms of how the children have handled the situation in Sweden. |
|  | Facilitation and restricting factors |  | Child-dependent factors:  Age, e.g. have knowledge.  Social situation, e.g. money  Social factors:  Adults wish or not wish to involve the child.  Examples of other family members and friends  Connections  Contextual factors:  Urgency, e.g., violence and oppression  Officials such as UNHCR  Chance | Child-dependent factors:  Age, e.g. have knowledge.  Social situation, e.g. money  Social factors:  Adults wish or not wish to involve the child.  Examples of other family members and friends  Connections  Contextual factors:  Urgency, e.g., violence and oppression  Officials such as UNHCR  Dependence on smugglers  Chance | Ownership of one's own life situation – take advantage of the opportunities that are given.  The agency can be restricted by society, e.g., by the appointed legal guardian but also by well-meaning, e.g., civil society. |
|  |  | Gender | - | - | - |
|  |  | Asylum status | Quota refugees do not make the decision on their own. | - | - |
| Reaching the full age can change everything |  |  | - | Whether or not to migrate is decided by the age of the individual. | The right to family reunification and education changes due to reaching the age of 18 years.    After reaching the full age, there are legal rights to control decisions and govern oneself. |
|  |  | Gender | - | - | - |
|  |  | Asylum status | Quota refugees have a different position. | Quota refugees have a different position. | Quota refugees have a different position.  Rights differ depending on the status. |
